# Better than expected: the gap between self-reported and objective measures of cognitive performance in remitted bipolar disorder

**DOI:** 10.3389/fpsyt.2023.1258303

**Published:** 2023-09-18

**Authors:** Esther Quinlivan, Babette Renneberg, Stefanie Schreiter, Eva Friedel, Olga Shmuilovich, Thomas Stamm

**Affiliations:** ^1^Department of Psychiatry and Neurosciences, Charité – Universitätsmedizin Berlin, Corporate Member of Freie Universität Berlin, Humboldt-Universität zu Berlin, and Berlin Institute of Health, Berlin, Germany; ^2^Clinical Psychology and Psychotherapy, Freie Universität Berlin, Berlin, Germany; ^3^Department of Psychiatry, Psychotherapy and Psychosomatic Medicine, Vivantes Klinikum Am Urban, Berlin, Germany; ^4^Department of Psychology, Brandenburg Medical School Theodor Fontane, Neuruppin, Germany; ^5^Schloss Luetgenhof Hospital, Centre for Personal Medicine, Psychosomatics and Psychotherapy, Dassow, Germany

**Keywords:** bipolar disorder, self-reported cognitive functioning, executive functions, objective cognitive functioning, self-esteem, euthymic, remitted bipolar disorder

## Abstract

**Background:**

Studies comparing objective and self-reported cognitive functioning as well as influencing factors in individuals with remitted bipolar disorder are scarce and contradictory.

**Methods:**

The aim of this study was to compare executive functioning and other objective and self-reported cognitive impairment between 26 individuals with remitted bipolar disorder (15 BD I) and 24 healthy controls using a cross-sectional design. Executive functions were measured by the TAP Go/No-go subtest as well as the Stroop Task. Self-rated functioning was assessed using the Attention Deficit Experience Questionnaire. In addition, possible predictors of self-reported and objective cognitive functioning were examined to perform regression analyses.

**Results:**

Individuals with remitted bipolar disorder did not differ significantly in executive functions or other objective cognitive domains from the healthy control group, but showed a significantly lower level of self-reported cognitive functioning and self-esteem. While self-esteem was the strongest predictor in healthy controls for self-reported cognitive functioning, severity of illness and subthreshold depressive mood were the most important predictors in individuals with remitted bipolar disorder.

**Conclusion:**

The results once again demonstrate the cognitive heterogeneity in bipolar disorder. In the treatment of cognitive deficits, factors such as subthreshold depressive symptomatology and self-esteem should be focused on in addition to cognitive training in remitted patients.

## Introduction

Impairment of neurocognitive functioning appears to be evident during acute mood episodes in individuals with bipolar disorder [BD; (1–3)]. But does this observation really apply to all phases of the disorder? Researchers have been striving for years to elucidate the underlying factors for impaired functioning and quality of life also in individuals with remitted BD (rBD). Lin et al. (2) found deficits in rBD undergoing cognitive tests persisting to a lesser degree in various cognitive domains. A meta-analysis (4) also demonstrated moderate cognitive impairment in rBD on a range of standard neuropsychological tests. The authors emphasize that current levels of mild depressive symptoms and effects of some drug treatments may contribute to these effects but do not explain them. Some studies have not only found deficits in cognitive functions in rBD, but rather observed a heterogeneous pattern consisting of mostly three subgroups: in addition to general cognitive impairment, subgroups with selective cognitive impairment and intact cognition were identified (5–7).

When discussing cognitive performance, self-assessment of cognitive function might play an important role besides objective measures: Lin et al. (2) found almost 3 out of 4 BD patients across all phases reporting significant self-reported dysfunction in relation to healthy controls (HC). Rosa et al. (8) showed significant correlations between subjective complaints and single objective cognitive measures related to executive function, working memory, verbal and visual memory in participants with rBD. However, self-assessed cognitive skills do not necessarily correlate with objectively measured cognitive abilities and studies observe controversial results (2, 9). Studies also showed that people with BD did not complain about their cognitive ability but showed impairment on neurocognitive tests (10, 11). Thus, using only subjective or objective measures seems limited in detecting cognitive impairment in BD (5, 12).

There are demographic and clinical factors that may influence the objective cognitive performance as well as the subjective evaluation of cognitive capabilities, such as age, IQ, years of education, medication, presence of childhood trauma, hospitalization, or number of episodes (5, 6, 8). The significant impact of sleep disorders and underlying depressive symptoms on cognitive deficits such as reduced speed, deficits in attention, and deficits in verbal memory were discussed (13). Mood changes seem to have a particular impact on self-reported cognitive impairment: several studies found depressive mood to be a predictor of subjective cognitive complaints in people with BD (14), but not as moderator between subjective and objective cognition (9).

Given that mood has a crucial influence on self-reported cognitive impairments, it seemed apparent, raising the question of self-esteem as a potential influencing variable. We already know that self-esteem is unstable in rBD compared to HC (15–17). However, what we do not yet know is whether and to what extent self-esteem is related to self-reported cognitive abilities in BD. In our study, we therefore wanted to investigate this exploratively.

We compared cognitive performance on objective neurocognitive tests focusing on executive functions, as well as self-reported cognitive abilities and related factors such as self-esteem in people with rBD and HC to further elucidate their relationship to each other. First and foremost, we hypothesized that individuals with rBD would show worse executive functioning than HC. Secondarily, we hypothesized that individuals with rBD would underestimate their cognitive performance and have lower self-esteem than HC. Furthermore, we explored possible correlations between objective cognitive performance and self-reported cognitive performance in both groups. In addition, we examined possible predictors such as clinical and demographic factors and self-esteem for self-reported cognitive performance. We additionally hypothesized that subthreshold mood symptoms would predict variance in self-reported cognitive performance.

## Materials and methods

### Participants and design

We chose a cross-sectional correlational design. All participants provided written informed consent. Inclusion criteria were:

- all participants were 18 to 65 years old
- participants had to be remitted for a minimum of 6 months prior to testing (excludes present rapid cycling) as determined by a structured clinical interview [SCID-I, (18)]
- only subthreshold current symptoms were allowed as defined by Bonnìn et al. (19) with a current score of ≤9 on the 21-items-version of the Hamilton Depression Rating Scale [HAMD; (20)] and a score of ≤12 on the Young Mania Rating Scale [YMRS; (21)]
- participants’ psychopharmacological treatment had to be stable for at least 6 preceding weeks (no switch and no dosage change during that period)

Exclusion criteria included:

- a history of substance abuse or electroconvulsive therapy in the past 6 months
- suicidal tendency
- ongoing in-patient-treatment
- a previous diagnosis of schizoaffective disorder, schizophrenia, antisocial personality disorder, dementia, mild cognitive impairment or mild intellectual disability, epilepsia, any neurogenerative disorder
- pregnancy or breastfeeding

Individuals with rBD were not preselected on the basis of any neurocognitive measure before entering the study. They underwent extensive neuropsychological and clinical assessments on two different days, 2 h per day. The control sample was recruited by online advertisement. Exclusion criteria for HCs included additionally a history of any mental disorder as assessed via the Mini International Neuropsychiatric Interview [MINI; (22)], self-reported severe physical health problems, and first-degree relatives with a mental disorder. Ethical approval was obtained from the Ethics Committee of the Charité - Universitätsmedizin Berlin.

26 healthy controls and 27 individuals diagnosed with bipolar disorder type I or II according to ICD-10 criteria were recruited at the psychiatric outpatient clinic of the Department of Psychiatry and Neurosciences at Charité University Hospital Berlin, Campus Mitte. One participant was excluded from analysis *post hoc* due to a depressive episode less than 6 weeks ago. Two healthy controls were excluded *post hoc* from the analysis due to a history of an affective disorder. The final clinical sample included 26 remitted bipolar individuals (15 with bipolar type I; 13 females, between 23 and 65 years old) and 24 healthy controls (16 females, between 19 and 63 years old).

### Materials

#### Clinical measures

In the bipolar group the Diagnostic and Statistical Manual of Mental Disorders (4th ed., DSM-IV) evaluated with SCID-I (18) was used to assess diagnosis and course of the disorder during the last 6 months and HAMD and Beck Depression Inventory [BDI; (23)] were used to assess current depressive symptoms. Manic symptoms were assessed with the YMRS and ASRM (24). Also, duration of illness and number of previous depressive and (hypo)manic episodes were recorded. Controls were administered by the MINI and the BDI. For further demographic characteristics see Table 1.

**Table 1:** Demographic and clinical characteristics of bipolar group and healthy control subjects.

Measures	rBD *n* = 26	HC *n* = 24	Statistics	*p*	*df*
Age in years, *M* (SD)	44.81 (11.98)	39.04 (13.55)	*t* = 1.597	0.117	48
Years of education, *M* (SD)	16.04 (2.39)	15.37 (2.81)	*t* = 0.901	0.372	48
Number of female participants (%)	13 (50%)	16 (67%)	*Χ^2^* = 1.423	0.233	1
BDI score, *M* (SD)	7.48 (7.52)	3.54 (3.22)	*t_w_* = 2.399	0.022*	32.77
HAMD score, *M* (SD)	4.60 (2.40)				
YMRS score, *M* (SD)	1.40 (2.40)				
ASRM score, *M* (SD)	2.60 (2.80)				
Bipolar Disorder type I, *n* (%)	15 (57.69)				
Number of hospitalizations, *M* (SD)	2.42 (2.25)				
Age at onset in years, *M* (SD)	24.32 (9.24)				
Number of episodes total, *M* (SD)	21.73 (14.63)				
depressive episodes, *M* (SD)	11.50 (6.99)				
hypomanic episodes, *M* (SD)	7.69 (9.17)				
manic episodes, *M* (SD)	2.50 (3.28)				
Number of psychiatric medication, *M* (SD)	2.16 (0.85)				
Mood stabilizers, *n* (%)	22 (84.62)				
Antipsychotics, *n* (%)	10 (38.46)				
Antidepressants, *n* (%)	10 (38.46)				
Anxiolytics, *n* (%)	3 (11.54)				

*p < 0.05 (indicating statistical significance); *t_w_*, Welche’s *t*-values; rBD, remitted bipolar individuals; HC, healthy controls; BDI, Beck Depression Inventory; HAMD, Hamilton Depression Rating Scale; YMRS, Young Mania Rating Scale; ASRM, Altman Self-Rating Mania Scale.

#### Neuropsychological tests

Selected aspects of executive functioning, attention, memory and general cognitive ability were measured using a total of 17 tests.

Executive functions were measured by the Stroop Colour-Word Task (25). In this very well-known test, participants have to look at a list of words printed in a different color than the meaning of the word. They are then asked to name the color of the word as quickly as possible, but not the word itself. We calculated the completion time to determine executive function in general. In addition, cognitive inhibition was measured with the “Go/ No-go” subtest of the computerized Test of Attentional Performance [TAP; (26)]. In this paradigm, subjects must direct attention to predictably occurring stimuli that require a selective response, i.e., to respond or not to respond.

To measure different aspects of attention, further subtests of the TAP were used. Tonic and phasic attention was measured with the subtest “Alertness”: in this test, subjects are shown a cue stimulus with a warning tone and the corresponding reaction time in response to the critical stimulus is measured.

The visual and auditory divided attention was measured with the subtest “Divided Attention” (reaction time): a visual and an auditory task must be processed in parallel. We measured the reaction time. General divided attention was assessed with the same subtest by the number of dropouts.

To assess memory functioning, we used subtests of the revised Wechsler Memory Scale WMS-R; (27): auditory short-term memory and auditory working memory were measured using the “Digit Span Forward” and “Digit Span Backward” subtests. In “Digit Span Forward,” a sequence of numbers is read out to the subject, who calls it up in the same order. In “Digit Span Backward,” a sequence of numbers is read aloud to the subject, who calls it out in reverse order.

To test the verbal learning, verbal consolidation and verbal recognition we used The Verbal Learning and Memory Test [VLMT; (28)]. VLMT requires the learning of word lists and thus enables the assessment of various memory parameters.

Verbal intelligence was measured with version B of the Mehrfachwahl-Wortschatz-Intelligenztest [MWT-B; (29)] which is similar to the Spot-the-Word Test (30). The MWT-B consists of a short instruction for the participant to find out if there are any of the presented words in each of the following lines.

Word fluency is considered as a factor of verbal intelligence. We measured phonemic and semantic verbal fluency with the subscales “S-words” and “animal names” of the Regensburger Wortflüssigkeits-Test [RWT; (31)].

The LPS 3 (32) is a German intelligence test based on Thurstone’s primary mental abilities. We used the third subtest to assess reasoning abilities: one of the eight characters in each line contradicts an underlying rule. The participant must recognize the rule and cross out the incorrect character.

#### Self-reported cognitive functioning and self-esteem

With the Questionnaire for Experiences of Attention Deficits [QED; (33, 34)] we assessed the subjective complaints and cognitive functioning in daily life. 27 items are assigned to the following subscales: (1) distractibility and deceleration of cognitive performance (cognitive dimension: QEDc), (2) fatigue and deceleration of practical tasks (practical dimension: QEDp), and (3) motivation level (motivational dimension: QEDm). The three subscales of QED allow us to explore different dimensions of self-perceived cognitive functioning in daily life and to set those in relation to possible predictors, respectively.

Participant’s self-esteem was operationalized using the German version of the Rosenberg Self-Esteem Scale [RSES; (35)].

#### Statistical analyses

The data was analyzed using SPSS version 25 (SPSS Inc., Chicago, IL, United States). To check whether the data exhibited a normal distribution the continuous variables were visually explored using histograms and box-plots. The outliers were identified and the assumption of normality was tested using the Shapiro–Wilk-Test within each group separately. In case of non-normally distributed variables, bootstrapping was applied as a resampling technique (with 2000 samples and the bias-corrected and accelerated bootstrap interval). In order to test group differences between rBD and HC the independent samples *t*-test was used for continuous variables and chi-square test was applied for categorical variables. In case of the violation of the homoscedasticity the Welche’s *t*-values were reported. Pearson correlations were used to quantify bivariate associations. As a default we report bootstrapped confidence intervals for r-coefficients. Linear regression analyses were carried out to explore possible predictors of self-reported cognitive performance. Based on the previous findings (2, 5, 36) following variables were selected as possible predictors in the rBD group: age (2, 36), years of education (2, 5), age at onset (2, 36), number of episodes as equivalent for duration of illness (2), depressive symptoms [BDI and HAMD scores, respectively; (2, 36)], manic symptoms [YMRS, (2)]. In addition, self-esteem (RSES) was exploratively analyzed as this is a new aspect in our study. Additionally, age at onset and self-esteem (RSES) were analyzed exploratively. In the HC group subthreshold depressive symptoms (BDI), age, years of education and self-esteem (RSES) were taken into account. Using the stepwise method, all candidate predictors were entered into the regression to further keep only those variables that are found to be statistically significant and to contribute uniquely to the regression equation.

The assumption of heteroscedasticity for multiple regressions was statistically tested using the Breusch-Pagan test and the Koenker test. Collinearity diagnostics were considered. The variance inflation factor (VIF) below 10 and the values of tolerance above 0.1 were considered adequate (37). Only the standardized beta weights are reported. All tests were two-tailed and statistical significance was defined as a value of *p* < 0.05. For the main outcome, we performed an *a priori* power analysis with G^*^Power, calculating a group size of *N* = 26 per group with an effect size of =0.80.

## Results

### Demographic and clinical characteristics

Our final study sample included 26 individuals diagnosed with BD and 24 HCs. Demographic and clinical features of the samples are displayed in Table 1. The groups did not show statistically significant differences in their demographic variables such as age, gender, and years of education. No individual diagnosed with BD was drug-free (see Table 1). 8 out of 26 rBDs (30.77%) presented comorbid psychiatric conditions (such as personality disorder, anxiety, past substance abuse or addiction). The use of alcohol, cannabis and other drugs (never/very rarely per month/several times a week/daily) as well as regular smoking (yes/no) were assessed in both groups. No group differences were found regarding the substance use or smoking (*p*’s ≥ 0.095).

### Executive functions

Following our first hypothesis, we looked for group differences in the executive functioning as a measure of objective performance between rBDs and HCs: in Go/No-go test the mean reaction times [*t*_w_ = 2.154, *df*_w_ = 37.475, *p* = 0.038, *d* = 0.06 (0.02; 1.16)] were significantly higher in the bipolar group (Table 2). However when controlling for age, the group difference in the Go/No-go test disappeared [*F* (1, 47) = 2.226, *p* = 0.142, *η* = 0.05], pointing out a significant interaction with group and age as a covariate [*F* (1, 47) = 12.066, *p* = 0.001, *η* = 0.20]. Further, there was a statistical trend in Stroop test regarding the higher mean completion time in the rBD group compared to HC [*t* = 1.938, *df* = 48, *p* = 0.055, *d* = 0.55 (− 0.02; 1.11)]. Other neurocognitive measures are reported in Table 2.

**Table 2:** Group comparisons for neurocognitive tests.

Measures	rBD (*n* = 26) *M* (SD)	HC (*n* = 24) *M* (SD)	Statistics	*p*	*df*
Stroop (RT)	80.64 (15.28)	72.39 (15.12)	*t* = 1.938	0.055	48
TAP: go/no-go (RT)	432.31 (97.70)	385.75 (49.04)	*t_w_* = 2.154	0.038^*^	37.47
TAP: go/no-go (number of errors)	1.08 (1.74)	0.58 (0.88)	*t* = 1.248	0.218	48
TAP: tonic alertness (RT)	44.81 (11.98)	39.04 (13.55)	*t* = 1.597	0.117	48
TAP: phasic alertness (RT)	−0.03 (0.08)	−0.05 (0.13)	*t* = 0.920	0.362	48
TAP: visual divided attention (RT)	789.61 (126.28)	792.16 (102.91)	*t* = −0.078	0.938	48
TAP: auditory divided attention (RT)	622.15 (132.41)	565.37 (87.73)	*t* = 1.771	0.083	48
TAP: divided attention (number of lapses)	1.65 (1.72)	1.58 (2.06)	*t* = 0.132	0.896	48
MWTB (total correct items)	30.69 (3.31)	29.79 (4.11)	*t* = 0.856	0.396	48
WMS-R: digit span forward (number of correct trials)	8.81 (1.65)	8.29 (2.25)	*t* = 0.929	0.358	48
WMS-R: digit span backward (number of correct trials)	6.84 (2.37)	7.37 (2.08)	*t* = −0.834	0.409	48
RWT: subtest animal names (total correct responses)	24.48 (6.58)	27.54 (5.80)	*t* = −1.530	0.133	48
rwt: subtest S-words (total correct responses)	14.42 (4.46)	16.50 (4.32)	*t* = −1.668	0.102	48
LPS3 (total correct items)	27.19 (5.08)	28.45 (6.12)	*t* = −0.788	0.434	48
VLMT (recognition)	13.69 (1.55)	13.36 (2.06)	*t* = 0.475	0.639	25
VLMT (consolidation)	2.07 (2.32)	0.93 (1.73)	*t* = 1.463	0.156	25
VLMT (learning)	53.19 (9.68)	56.04 (9.06)	*t* = −1.974	0.288	48

*p < 0.05 (indicating statistical significance); *t_w_*, Welche’s *t*-values; RT, reaction times; rBD, remitted bipolar individuals; HC, healthy controls; TAP, Test of Attentional Performance; MWTB, Mehrfach-Wortschatz-Intelligenztest; WMS-R, revised Wechsler Memory Scale; RWT, Regensburger Wortflüssigkeits-Test; LPS3, Leistungsprüfsystem third subtest; VLMT, The Verbal Learning and Memory Test.

### Self-reported cognitive functioning and self-esteem

As hypothesized, individuals diagnosed with BD presented significantly lower QED scores as compared to the control group on all three subscales (Table 3). rBDs also showed a significantly lower self-esteem rating than HC (Table 3). When controlling for sex and age the group statistics in RSES and QED did not change (*p* ≤ 0.004).

**Table 3:** Differences in the group means of self-reported cognitive functioning and self-esteem.

Variables	rBD *M* (SD)	HC *M* (SD)	Statistics *t_w_*	*p*	*df_w_*	*d* [CI]
QEDc	46.79 (10.02)	57.96 (4.05)	−5.239	0.000***	33.48	−1.44 [−2.06; −0.81]
QEDp	30.81 (6.76)	37.37 (2.82)	−4.543	0.000***	34.04	−1.25 [−1.85; −0.63]
QEDm	21.46 (5.77)	26.50 (2.88)	−3.948	0.000***	37.42	−1.09 [−1.68; −0.59]
RSES	22.38 (6.29)	26.97 (3.38)	−3.248	0.001**	39.01	−0.89 [−1.47; −0.31]

Cohen’s *d*: very small (0.10), small (0.20), medium (0.50), large (0.80), very large (1.20), huge (2.00); [CI] = 95% confidence interval; *p < 0.05 (indicating statistical significance), **p < 0.01, ***p < 0.001; *t_w_* = Welche’s *t*-values; rB, remitted bipolar individuals; HC, healthy controls; QEDc, ognitive dimension of Questionnaire for Experiences of Attention Deficits; QEDp, ractical dimension of Questionnaire for Experiences of Attention Deficits; QEDm, motivational dimension of Questionnaire for Experiences of Attention Deficits; RSES, Rosenberg Self-Esteem Scale.

### Associations between self-reported and objective cognitive functioning and other variables

In both groups we did not find any significant correlations between self-reported cognitive functioning as measured by the QED subscales and the neurocognitive test results (all *p*’s > 0.101). Other correlations between the QED subscales and further variables of interest are presented in Table 4.

**Table 4:** Associations between self-reported cognitive functioning (three QED subscales) and other selected variables of interest.

	QEDc		QEDp		QEDm	
Measures	rBD	HC	rBD	HC	rBD	HC
	*r* [CI]	*r* [CI]	*r* [CI]	*r* [CI]	*r* [CI]	*r* [CI]
RSES	0.344 [−0.053; 0.668]	0.236 [−0.117; 0.521]	0.405 [−0.059; 0.798]	0.658*** [0.330; 0.845]	0.722*** [0.394; 0.930]	0.491* [0.173; 0.823]
BDI	−0.487* [−0.762; −0.113]	−0.225 [−0.552; 0.113]	−0.170 [−0.525; 0.177]	−0.502* [−0.743; −0.141]	−0.393 [−0.718; −0.051]	−0.540** [−0.782; −0.231]
HAMD	−0.206 [−0.585; 0.313]		−0.402 [−0.666; 0.061]		−0.485* [−0.779; −0.066]	
YMRS	−0.352 [−0.694; 0.106]		0.133 [−0.177; 0.394]		−0.036 [−0.402; 0.449]	
Age at onset	0.166 [−0.172; 0.478]		0.086 [−0.259; 0.485]		0.348 [0.023; 0.615]	
Number of hospitalizations	−0.102 [−0.485; 0.317]		0.024 [−0.413; 0.321]		0.043 [−0.365; 0.459]	
Number of episodes	−0.611** [−0.823; −0.297]		−0.427* [−0.740; −0.116]		−0.068 [−0.417; 0.294]	

*p < 0.05 (indicating statistical significance), **p < 0.01, ***p < 0.001; [CI] = 95% confidence interval; rBD, remitted bipolar individuals; HC, healthy controls; BDI, Beck Depression Inventory; HAMD, Hamilton Depression Rating Scale; YMRS, Young Mania Rating Scale; QEDc, cognitive dimension of Questionnaire for Experiences of Attention Deficits; QEDp, practical dimension of Questionnaire for Experiences of Attention Deficits; QEDm, motivational dimension of Questionnaire for Experiences of Attention Deficits; RSES, Rosenberg Self-Esteem Scale.

### Prediction of self-reported functioning

In the rBD group the best prediction model for QEDc (*F* = 10.818, R^2^ad = 0.483, Durbin-Watson = 2.078, *p* < 0.001; VIF max = 1.024, Tolerance min = 0.977) included the number of episodes and the BDI scores (see Table 5). Thus, 45% of the variance in self-reported cognitive functioning was accounted for by the number of episodes and the depressive symptoms, uniquely. In the second regression model only self-esteem (RSES score) proved itself as a significant predictor of the QEDm and explained almost 50% of its variance (*F* = 21.716, R^2^ad = 0.497, Durbin-Watson = 2.040, *p* < 0.001). Further, the number of episodes contributed significantly to the prediction of QEDp in the third regression model for rBDs (*F* = 5.343, R^2^ad = 0.148, Durbin-Watson = 1.820, *p* < 0.05).

**Table 5:** Regression coefficients of the regression analyses.

Dependent variables	Predictors	*β*	*p*	*r_part_*
Remitted bipolar individuals				
QEDc	Number of episodes Depressive symptoms (BDI)	−0.549 −0.404	0.001** 0.010*	−0.543 −0.399
QEDp	Number of episodes	0.427	0.030*	
QEDm	Self-esteem (RSES)	0.722	0.000***	
Healthy controls				
QEDp	Self-esteem (RSES)	0.658	0.000***	
QEDm	Depressive symptoms (BDI)	−0.540	0.006**	

*p < 0.05 (indicating statistical significance), **p < 0.01, ***p < 0.001; *β* = standardized beta; *r_part_* = semi-partial correlation; QEDc cognitive dimension of Questionnaire for Experiences of Attention Deficits; QEDp, practical dimension of Questionnairefor Experiences of Attention Deficits; QEDm: motivational dimension of Questionnaire for Experiences of Attention Deficits; BDI, Beck Depression Inventory; RSES, Rosenberg Self-Esteem Scale; HAMD, Hamilton Depression Rating Scale.

In the HC group, only RSES proved itself as a significant predictor of the QEDp (Table 5), explaining 40% of its variance (*F* = 16.768, R^2^ad = 0.407, Durbin-Watson = 2.785, *p* < 0.001). In contrast, QEDm in HC was significantly predicted by the BDI score (*F* = 9.073, R^2^ad = 0.260, Durbin-Watson = 1.527, *p* = 0.006). For QEDc no significant contributors were found in the HC group.

## Discussion

In this study, we compared people with rBD and HCs regarding their executive functioning and self-reported cognitive performance and related factors. To our knowledge, this is the first study investigating self-esteem as a possible predictor of cognitive functioning in rBD.

Surprisingly, there was no group difference between rBC and HC in executive functions or in the other neurocognitive functions we examined. This is noteworthy in that previous studies often observed group differences between rBDs and HC (2, 4, 38, 39). But on the other hand, there are also findings in which rBDs showed deficits neither in the area of executive functions nor in other cognitive domains (5–7, 13, 40). However, these were studies with a larger sample size, which allowed clustering of subgroups. Reviewing studies using neuropsychological tests similar to ours and focusing on executive functions such as inhibitory control or delay of reward as markers of impulsivity, a heterogeneous picture also emerges: for behavioral tests as markers of impulsivity such as the Go/No go Task, Stroop Test or Continuous Performance Task, there is little but some evidence of significant deficits in euthymic bipolar patients (41, 42). On the other hand, impulsivity measured by self-report has been consistently shown to be significantly increased in bipolar patients. Nevertheless, no correlation has yet been found between impulsivity measured by behavioral tests (as in our study) and impulsivity measured by self-report (42).

As expected, rBD participants underestimated their cognitive performance. This is in line with previous investigations (2, 8). As mentioned above, group statistics did not change when controlling for age and gender, which is different from Lin et al. (2), who observed bipolar individuals with higher levels of subjective cognitive dysfunction more likely to be female.

This study is the first to examine self-esteem in relation to objective and self-reported cognitive functioning in BD. In our sample, individuals with rBD showed lower self-esteem compared to HCs, as previously shown (15). This can be interpreted in the context of vulnerabilities and challenges such as stigma and exclusion faced by individuals with BD. Self-esteem is an interesting construct and might function as a possible contributor in the intermediation of self-reported and objectively measured cognitive performance in BD. It is reasonable to assume a reciprocal influence between self-esteem and cognitive performance. Most importantly, we find self-reported but no objective cognitive deficits in individuals with rBD compared with HC. Our results address the assumption that rBD do not necessarily face cognitive deficits but rather do experience distress in this regard (2). Furthermore, they underline previous findings in which self-reported and objectively measured cognitive functions did not correlate with each other (9–11).

Exploring possible predictors for cognitive functioning, we found solid predictors for self-reported cognitive functioning in our sample. We found that self-esteem is an important factor in assessing one’s perception and evaluation of cognitive performance. Self-esteem contributed to a significant variance explanation of self-reported cognitive performance in both people with rBD and healthy controls. These results reemphasize the important role of self-esteem in the context of cognitive performance. As expected, underlying mood issues appear to be another important predictor.

In this regard, the results are consistent with previous findings (2, 5, 8, 12). It has been argued that self-reported cognitive difficulties reflect mood better than actual, objectively measured cognitive performance (2). In contrast to previous research, in our rBD sample, the number of episodes was the strongest predictor of the cognitive dimension of self-reported cognitive performance. Possibly, the number of episodes reflects the severity of the condition and its impact on self-perceived cognitive performance.

We consider the small sample size, albeit sufficiently powered, as one of the main limitations in our study. It is mainly the group size that prevents us from forming a more differentiated picture including subgroups regarding the cognitive performance of the BD participants as many other research groups have done (5–7, 40). Looking at the statistical analyses, we did not correct for a possible familywise error rate across the reported statistical analyses because there is little consensus among experts on the appropriate degree of correction (43, 44). Therefore, we tried to keep the number of the statistical analyses as minimal as possible and encourage replications with greater sample sizes. The influence on self-reported and objective performance due to medication effects could not be controlled for. All of our participants in the rBD group received psychiatric medication. Pharmacological treatment like antidepressants or antipsychotics could affect patients´ self-reported and objective cognitive performance (45). In our study sample, the comparatively long interval to the last episode of 6 months is a methodological strength. Future studies need to show which pattern emerges with a larger study sample and similarly long interval to the last acute episode. In addition, it would be pioneering to conduct further studies of this type in a longitudinal design, as Volkert et al. (13) have already done.

Our results invite us to take a more nuanced view on bipolar disorder and its challenges. Objective impairments in cognitive function do not appear to persist across all phases of the disorder, as has been argued elsewhere (46). They are not a characteristic of rBD. Clinicians may consider testing the cognitions of individuals with BD only in remission and after an appropriately long interval from the last acute episode. Only then can a statement be made about possible impairment of neurocognitive performance. Asides from that, potential confounding factors such as sleep or medication appear to have an influence. Moreover, self-reported cognitive impairment appears to be influenced by factors such as residual symptomatology and self-esteem. For people with BD who continue to have cognitive impairment even after remission of an acute episode, cognitive training should be a component of treatment. Furthermore, within psychiatric and psychotherapeutic treatment, it would be beneficial to pay attention to factors such as self-esteem or the impact of previous episodes on self-evaluation, in addition to residual depressive symptoms.

## Data availability statement

The raw data supporting the conclusions of this article will be made available by the authors, without undue reservation.

## Ethics statement

The studies involving humans were approved by Ethikkommission der Charité Universitätsmedizin Berlin. The studies were conducted in accordance with the local legislation and institutional requirements. The participants provided their written informed consent to participate in this study.

## Author contributions

EQ: Writing – original draft, Writing – review & editing. BR: Supervision, Writing – review & editing. SS: Data curation, Writing – review & editing. EF: Writing – review & editing. OS: Data curation, Methodology, Writing – original draft. TS: Project–administration, Supervision, Writing – review & editing.

## Funding

The author(s) declare that no financial support was received for the research, authorship, and/or publication of this article.

## Conflict of interest

The authors declare that the research was conducted in the absence of any commercial or financial relationships that could be construed as a potential conflict of interest.

## Publisher’s note

All claims expressed in this article are solely those of the authors and do not necessarily represent those of their affiliated organizations, or those of the publisher, the editors and the reviewers. Any product that may be evaluated in this article, or claim that may be made by its manufacturer, is not guaranteed or endorsed by the publisher.

## Figures and Tables

**Table:** 

BD	Bipolar Disorder
rBD	remitted Bipolar Disorder
HC	healthy controls
HAMD	Hamilton Depression Rating Scale
YMRS	Young Mania Rating Scale
BDI	Beck Depression Inventory
ASRM	Altman Self-Rating Mania Scale
MINI	Mini International Neuropsychiatric Interview
LPS3	Leistungsprüfsystem
MWTB	Mehrfachwahl-Wortschatz-Intelligenztest
RWT	Regensburger Wortflüssigkeits-Test
VLMT	Verbal Learning and Memory Test
WMS-R	revised Wechsler Memory Scale
TAP	Test of Attentional Performance
QED	Questionnaire for Experiences of Attention Deficits
QEDc	cognitive dimension of QED
QEDp	practical dimension of QED
QEDm	motivational dimension of QED
RSES	Rosenberg Self-Esteem Scale
